# Isolated Medial Dislocation of Elbow: A Case Report

**DOI:** 10.31729/jnma.6669

**Published:** 2021-08-31

**Authors:** Kapil Mani KC, Ramesh Aryal, Samir Acharya, Amuda KC

**Affiliations:** 1 Department of Orthopedics, Mercy City Hospital, Butwal, Rupandehi; 2 Department of Anesthesiology, Mercy City Hospital, Butwal, Rupandehi, Nepal; 3 Department of Medical and Surgical Nursing, Nepalese Army Institute of Health Sciences, Sanobharyang, Kathmandu, Nepal

**Keywords:** *closed reduction*, *elbow*, *functional outcomes*, *isolated medial dislocation*

## Abstract

Even though posterior or postero-lateral dislocations of elbow are more common, both isolated medial and lateral dislocations of elbow are extremely uncommon. Since there are subtle findings and minimum pain after medial dislocation of elbow, these are sometimes missed by attending physician at first presentation and changes into chronic type with guarded prognosis. We report a case of a 15-year old boy with isolated medial dislocation of elbow which was correctly identified and treated with closed reduction and posterior slab application. Flexion extension as well as supination pronation of elbow 3 months after injury was nearly normal.

## INTRODUCTION

Simple elbow dislocations typically occur in the posterior or postero-lateral direction while isolated lateral and medial dislocations are extremely rare.^1^ Regarding the isolated medial dislocation of elbow, deformity is subtle so that either patients do not seek medical advice immediately because of lack of significant pain or findings might be missed by attending physician at first presentation.^2^ Therefore, simple dislocation may be changed into chronic type where prognosis becomes very guarded.

## CASE REPORT

**Figure 1:**
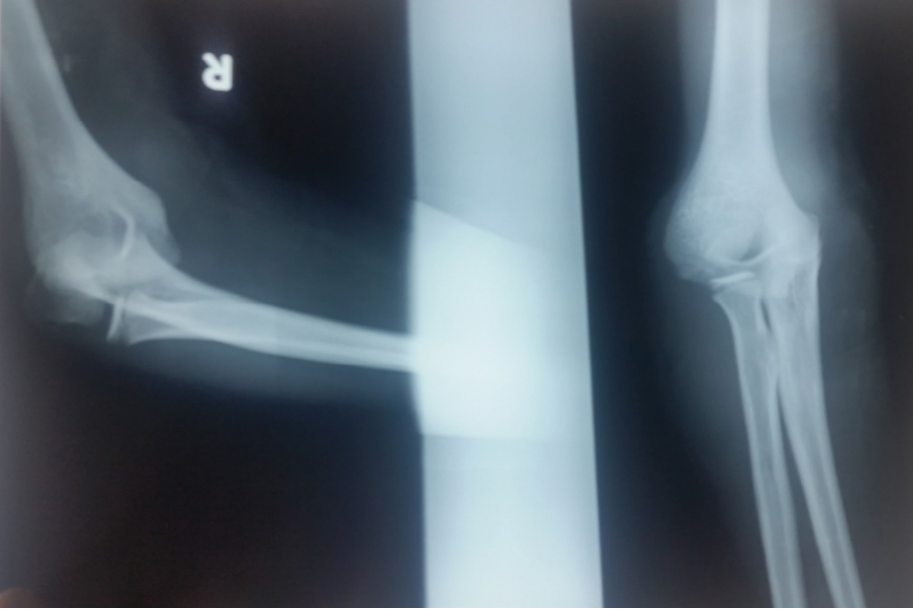
X-ray of right elbow isolated showing medial dislocation of right elbow.

A 15-year-old male patient came to our hospital with history of trivial injury on right elbow while playing football on the ground. On examination there was mild swelling, minimum deformity, and terminal restriction of flexion extension as well as supination pronation of elbow with intact distal neurovascular status. He was advised for X-ray right elbow joint antero-posterior and lateral views. X-ray showed the isolated medial dislocation of elbow without any associated fractures of distal humerus, proximal radius and ulna (Figure 1).

**Figure 2:**
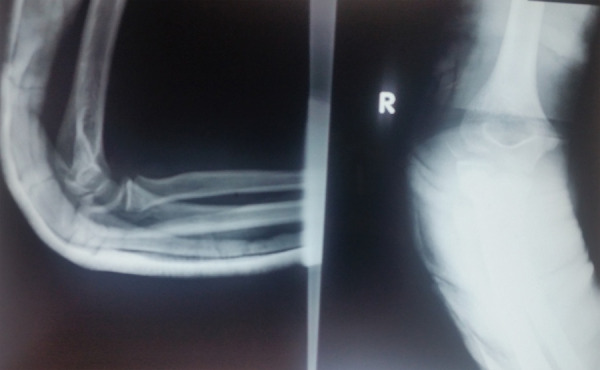
Post reduction X-ray of same elbow with application of posterior slab.

Elbow was reduced in emergency department under sedation in supine position with 90 degree bending of elbow giving traction and counter traction along with valgus and lateral thrust force on elbow (Figure 2).

Neurovascular status of limb was assessed after dislocation was reduced. Posterior slab was applied for 10 days and after that elbow mobilization exercise was started. Patient was followed up in OPD every second week and final range of motion of elbow was assessed 3 months after injury. Flexion extension was in the range of 130 degree to 10 degree while supination pronation was in the range of 80 to 75 degree.

## DISCUSSION

Elbow dislocations are classified by the direction of displacement and the presence or absence of associated fractures. Simple elbow dislocations are solely soft tissue injuries. The most common dislocation involves posterior (postero-lateral followed by postero-medial). Lateral and medial dislocations are extremely rare.^3^ Isolated medial dislocation of elbow is a rare condition, but it does occur. Saxena et al^3^ described that high clinical suspicion and good quality X-rays are required for early and prompt diagnosis. As with other dislocations, immediate reduction of the dislocated joint and splintage is all that required for proper soft tissue healing and range of motion retrieval. Aroojis et al^4^ mentioned an isolated medial dislocation of elbow in 10.5 years old child which was reduced under general anesthesia. At 4-year follow up, radiographs showed a normal alignment of the elbow with mild changes of heterotopic ossification. They believe that that was the first such case ever reported in literature. Early recognition and prompt diagnosis is the key to achieve a good result.

Jockel et al^1^ described 4 cases of simple medial elbow dislocations from 2000 to 2011 treated at a single referral center. Two elbows had been performed immediate closed reduction, and 2 elbows could not be reduced acutely. All patients had surgical repair of the lateral collateral ligament complex and extensor tendon origin. They concluded that simple medial elbow dislocations may be at risk for early instability and may represent a more noteworthy soft tissue injury than typical dislocation patterns. Surgical treatment of early instability in these injuries led to acceptable patient outcomes. Similarly Ohno, et al.^2^ quoted the chronic unreduced medial dislocation which is also an extremely rare injury.

We strongly recommend high index of suspicion, early identification, prompts intervention to achieve the good functional outcomes in relatively simple but very uncommon case of medial elbow dislocation which, if missed at early stage, has very guarded prognosis.
